# Archaea show different geographical distribution patterns compared to bacteria and fungi in Arctic marine sediments

**DOI:** 10.1002/mlf2.70006

**Published:** 2025-04-24

**Authors:** Jianxing Sun, Hongbo Zhou, Haina Cheng, Zhu Chen, Yuguang Wang

**Affiliations:** ^1^ School of Minerals Processing and Bioengineering Central South University Changsha China; ^2^ Key Laboratory of Biohydrometallurgy of Ministry of Education Changsha China

**Keywords:** Arctic Ocean, biogeography, co‐occurrence pattern, marine microorganisms, spatial distribution

## Abstract

Microorganisms dominate marine environments in the polar oceans and are known to harbor greater diversity and abundance than was once thought, and yet, little is known about their biogeographic distribution patterns in marine sediments at a broad spatial scale. In this study, we conducted extensive sampling of marine sediments along a latitudinal transect spanning 2500 km from the Bering Sea to the Arctic Ocean to investigate the geographical distribution patterns of bacteria, archaea, and fungi. Our findings revealed that the community similarities of bacteria and fungi decay at similar rates with increasing geographical distance (slope: −0.005 and −0.002), which are much lower than the decay rate of archaeal communities (slope: −0.012). Notably, microbial richness and community composition showed significant differences in the region of 75−80°N compared to other regions in 60−75°N. Salinity, temperature, pH, ammonium nitrogen, and total organic carbon are key factors that significantly affect microbial community variations. Furthermore, bacterial co‐occurrence networks showed more complex interactions but lower modularity than fungal counterparts. This study provides crucial insights into the spatial distribution patterns of bacteria, archaea, and fungi in the Arctic marine sediments and will be critical for a better understanding of microbial global distribution and ecological functions.

## Impact statement

Understanding the biogeography of marine microbes is essential for grasping biogeochemical cycling and climate regulation. This study on the Arctic marine sediments revealed distinct geographic distribution patterns of bacteria, archaea, and fungi. Archaea showed the strongest distance–decay pattern, while bacteria and fungi showed similar distributions. These findings advance our understanding of benthic microbial biogeography and the mechanisms driving microbial distribution. This knowledge is crucial for predicting microbial responses to rapid Arctic warming and developing strategies to preserve biodiversity and ecological functions, thereby ensuring the stability of marine environments globally.

## INTRODUCTION

Microorganisms are key components of marine ecosystems, playing irreplaceable ecological roles due to their sheer diversity, immense abundance, and diverse functions^1^, ^2^, ^3^. Understanding the biogeographical distribution patterns of marine microorganisms is one of the most important objectives of ecological research, as it provides insights into the mechanisms sustaining global microbial diversity^4^, ^5^, ^6^, ^7^. Recent studies have significantly advanced our understanding of marine microbial biogeography^8^, ^9^, particularly highlighting that microbial spatial distribution patterns showed strong links with environmental gradients. For instance, many studies revealed that environmental factors such as latitude, depth, temperature salinity, and nutrient availability are the major factors that impact global microbial biogeography^9^, ^10^, ^11^, ^12^, ^13^, ^14^, ^15^. Additionally, research using the *Tara Ocean* dataset has provided insights into microbial global biogeography and their functional traits^8^, ^16^, highlighting the spatial heterogeneity in microbial distribution and functional potential. The latest machine learning‐based predictive model indicates that environmental changes due to anthropogenic climate change will result in alterations in the ecological status and geographic distribution of surface ocean microorganisms for 32.4% of the surface ocean^17^. We have gained a substantial understanding of the regional and even global distribution patterns of marine microorganisms, revealing significant geographic differences in microbial diversity and distribution in seawater^18^, ^19^, ^20^. However, challenges such as limited access to ice‐breakers, insufficient funding, and scarce dynamic information about marine systems constrain our understanding of microbial diversity and geographic distribution in large‐scale marine sediments, particularly in polar regions.

Marine sediments represent a unique ecosystem distinct from the water column^21^, ^22^, ^23^. These sediments harbor a higher microbial diversity and abundance than overlying waters^19^, ^24^, playing important roles in nutrient cycling and biochemical processes^2^, ^25^, ^26^. In recent years, research on the diversity and distribution patterns of microorganisms in marine sediments has increased, revealing that the geographical distribution of microorganisms in marine sediments showed strong spatial heterogeneity^27^. For instance, studies on deep‐sea and coastal sediment microorganisms found that community compositions varied significantly in different oceanic regions^28^, ^29^. Metagenomic analyses of sediment samples from different regions further revealed pronounced spatial differences in microbial communities across ocean basins^30^, ^31^, ^32^. Despite these findings, current studies are often limited to specific regions with few samples, lacking systematic, large‐scale comparisons, especially in the Arctic Ocean^33^, ^34^, ^35^. Over the past decades, the Arctic has experienced rapid warming at rates 2 to 4 times faster than the global average^36^, ^37^, ^38^, posing significant threats to its ecosystems and raising concerns about the impact on microbial spatial distributions. Understanding microbial responses to climate change has become a critical focus, as evidence suggests that warming is a major threat to microbial diversity and community structure^39^, ^40^, ^41^, ^42^. However, current knowledge of microbial biogeographical patterns, particularly in polar oceans under changing conditions, remains limited. Additionally, existing research often fails to compare the distribution patterns of different microbial groups, such as bacteria, archaea, and fungi. Thus, comprehensive research is urgently needed to explore these patterns and understand spatial differences among microbial communities, especially under rapidly changing Arctic conditions.

In the realm of investigating microbial biogeography, co‐occurrence networks have emerged as a robust analytical tool for elucidating the complex interactions^43^ and ecological dynamics within microbial communities in various habitats^44^, ^45^, ^46^, ^47^, ^48^. Fundamentally, co‐occurrence networks are constructed based on co‐presence patterns of species, often operational taxonomic units (OTUs), wherein nodes signify distinct microbial entities and edges denote co‐occurrence interactions^49^. Additionally, co‐occurrence networks provide insights into positive or negative associations among microbial taxa, thereby unraveling fundamental ecological dynamics such as mutualism, competition, and niche partitioning governing microbial assemblages. Therefore, this methodological approach can offer a fresh lens through which to probe the intricate tapestry of microbial distributions across a spatial spectrum^50^, ^51^, ^52^. Unfortunately, although co‐occurrence network analysis has been widely applied to explore the co‐occurrence patterns of different microbial populations in various habitats^53^, ^54^, ^55^, it is still unclear whether bacteria, archaea, and fungi in polar marine sediments show similar or different co‐occurrence patterns.

In this study, marine sediments were sampled from the Arctic marines (including the Bering Sea, the Bering Strait, the Chukchi Sea, and the Arctic Ocean). Based on high‐throughput sequencing, we mainly aim to determine (i) how bacteria, archaea, and fungi distribute along latitude; (ii) the key factors that exert significant effects on microbial distribution; and (iii) whether microbial co‐occurrence network patterns among bacteria, archaea, and fungi vary in different geographical regions. These results would provide useful insights into the microbial geographical distribution of marine sediment at a regional scale.

## RESULTS

### Sediment characteristics among sampling sites

A total of 72 sediment samples were collected from the pan‐Arctic Ocean, covering a geographic distance of nearly 2500 km (sampling sites and related physicochemical information are provided in Table S1). Furthermore, our results also demonstrated that most environmental variables significantly changed with latitude (Figure S1). Specifically, the fitting analysis of physicochemical parameters and latitude showed that when the latitude is below 75°N, the sampling depth remains relatively constant (average 196 mbsl), while above 75°N, the depth increases significantly (average depth 1017 mbsl). In addition, depth, salinity, and pH significantly increased with latitude (*p* < 0.05), and these variables maximized in the region of 75−80°N. Conversely, temperature and the contents of total organic carbon (TOC) were strongly and negatively correlated with latitude (*p* < 0.01), and the contents of nitrate ion (NO_3_^−^) and ammonium ion (NH_4_^+^) also showed declining patterns along latitude, although these changes were not significant (Figure S1E,F).

### Microbial diversity in sediments

**Figure 1:**
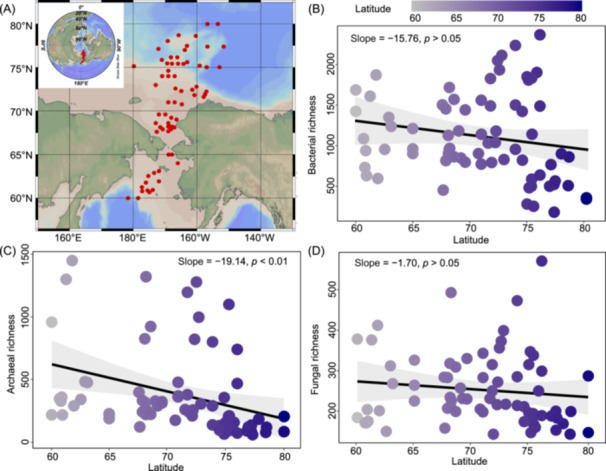
Map of sampling sites and microbial richness distribution along latitude. (A) Sampling sites in the pan‐arctic oceans (The maps were created using Ocean Data View software (ODV 4.7.7)). (B−D) Microbial richness of bacteria (B), archaea (C), and fungi (D), respectively. Linear regression lines and their 95% confidence limits (shaded gray area) are shown.

Microbial diversity (represented by richness and the Shannon index) showed different distribution patterns among bacteria, archaea, and fungi, even though all microbial diversity generally decreased with increasing latitude (Figures 1B–D and S1). The Mantel test indicated that archaeal richness significantly decreased with latitude (*p* < 0.05, Figure 1C), while this tendency was not significant in bacteria and fungi (*p* > 0.05, Figure 1B,D). The variation of the Shannon index was highly consistent with that of microbial richness (Figure S2). To explore microbial richness distribution patterns more accurately, we divided the whole area into four latitudinal regions every 5°N gradient (60–65°N, 65–70°N, 70–75°N, and 75–80°N). The results indicated that bacterial richness and Shannon diversity peaked at the 70–75°N region, whereas this phenomenon was not observed in archaea and fungi (Figure S3). Statistical analysis results indicated that there were no significant differences in bacterial alpha diversity when the latitude was lower than 75°N (Wilcoxon test, *p* > 0.05, Figure S3A,B), whereas the archaeal richness in the 75−80°N region was significantly lower compared to the other regions (*p* < 0.05, Figure S3C). Fungal alpha diversity in 65–70°N and 70–75°N was also significantly higher than that in the 75–80°N region (Figure S3E,F). Furthermore, almost all microbial diversities showed negative correlations with salinity (*p* < 0.01) and pH (*p* < 0.01) but positively correlated with NH_4_^+^ (*p* < 0.05) (Figure S4).

### Microbial distance–decay relationship and environmental influence

**Figure 2:**
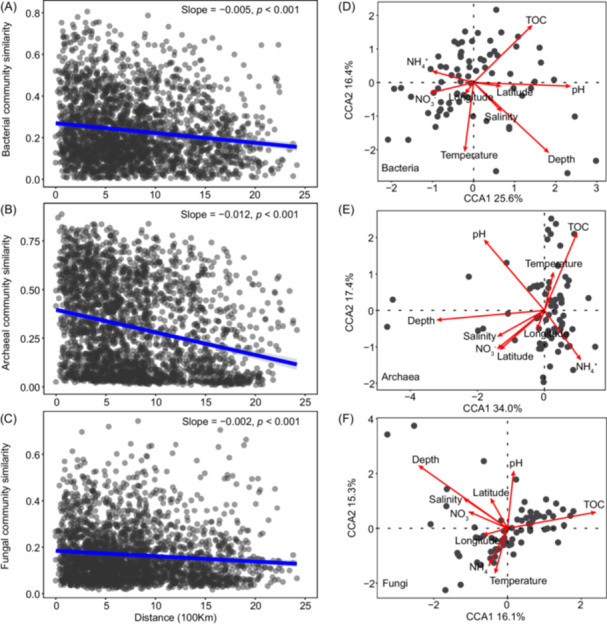
Distance–decay relationship and relationships between microbial community structures and environmental factors. (A–C) Community similarity distance–decay relationships for bacteria (A), archaea (B), and fungi (C), respectively. The solid blue line indicates the fit between geographic distance and Bray–Curtis similarity. The gray shadow under the solid line represents 95% confidence intervals around the linear model. (D–F) Ordination plot based on canonical correspondence analysis (CCA) of the relationships between microbial communities and environmental variables for bacteria (D), archaea (E), and fungi (F), respectively. Depth, water depth; TOC, total organic carbon.

Bacterial, archaeal, and fungal community similarities showed decreased tendency with geographical distance (Figure 2A–C), and a steeper slope was observed in the archaeal community (slope = −0.012, *p* < 0.001; Figure 2B) than that in bacterial (slope = −0.005, *p* < 0.001; Figure 2A) and fungal (slope = −0.002, *p* < 0.001; Figure 2C) communities. The overall variability of microbial distribution patterns of bacteria and archaea was analyzed by nonmetric multidimensional scaling (NMDS) analysis (Figure S5). Results of NMDS demonstrated that the regions of 60–65°N and 65–70°N generally clustered together and always clustered apart from other regions. Additionally, three different statistical tests indicated that the community structure differed significantly between the region of 75–80°N and other regions (Table S2), especially for the archaeal community. Furthermore, canonical correspondence analysis (CCA) indicated that all selected environmental variables together explained 42.0%, 51.4%, and 31.4% of bacterial (Figure 2D), archaeal (Figure 2E), and fungal (Figure 2F) community variation, respectively. In addition, analysis of the correlation with environmental factors based on the Mantel test revealed that the community composition of bacteria was significantly influenced by latitude, temperature, salinity, and TOC (*R* = 0.101 to 0.491, *p* < 0.05, Table S3), the community composition of archaea was significantly influenced by latitude, temperature, salinity, pH, and TOC (*R* = 0.070 to 0.086, *p* < 0.05, Table S3), while that of fungi was only significantly influenced by latitude and temperature (*R* = 0.159 and 0.361, *p* < 0.05, Table S3). In addition, variation partitioning analysis (VPA) results indicated that much higher community heterogeneity could be explained by purely environmental factors (10.1%–12.5%) than by spatial factors independently (5.9%–8.5%). A total of 5.5% of archaeal community variation could be explained by spatial and environmental factors together, but only 2.4% and 0.4% of bacterial and fungal community variations were explained by spatial and environmental factors together, respectively (Figure S6).

### Latitudinal variations in microbial community composition and environmental influences

**Figure 3:**
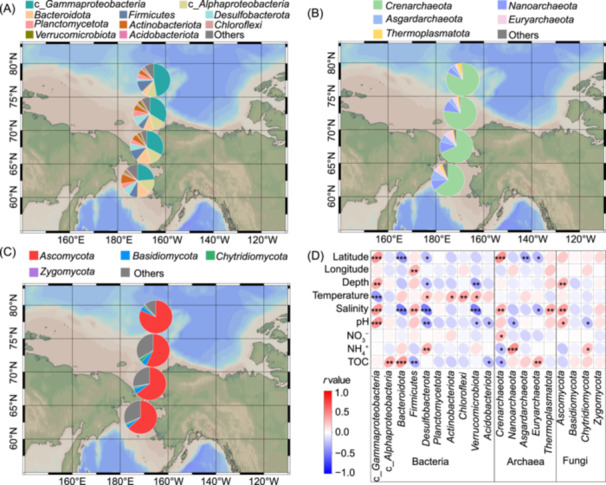
Main microbial community composition and its latitudinal distribution and the influence of environmental factors. (A–C) Community composition distribution along the latitudinal gradient regions of bacteria (A), archaea (B), and fungi (C), respectively. The maps were created using Ocean Data View software (ODV 4.7.7). (D) Environmental associations of the relative abundance of microbial phyla evaluated based on Spearman's correlation. The color gradient on the left indicates Spearman's rank correlation coefficients. ***, **, and * represent a statistically significant difference of *p* < 0.001, *p* < 0.01, and *p* < 0.05, respectively.

Microbial community compositions of these four latitudinal gradient regions at the phylum level (phylum *Proteobacteria* was divided into the classes *Alphaproteobacteria* and *Gammaproteobacteria*) are shown in Figure 3A–C. The results showed that the relative abundances of class *Gammaproteobacteria* and phylum *Crenarchaeota* gradually increased with latitude (Figure 3A,B), while the abundances of *Actinobacteriota* and *Asgardarchaeota* showed decreasing trends. Furthermore, when the latitude is over 75°N, the relative abundances of most species, such as class *Alphaproteobacteria*, *Bacteroidota*, *Desulfobacterota*, *Nanoarchaeota*, and *Euryarchaeota*, significantly decreased, whereas the abundances of *Firmicutes*, and *Thermoplasmatota,* significantly increased (Figure 3A,B). The abundance of *Ascomycota* was relatively stable (56.7%–64.7%) when the latitude was below 75°N, but its abundance increased to 80.6% in the region of 75–80°N (Figure 3C). The correlation between microbial taxa and environmental factors based on Spearman's correlation indicated that, compared to spatial factors, environmental factors had a more significant impact on microbial communities (Figure 3D), which is consistent with the results of the VPA analysis. Moreover, these results further revealed that latitude, depth, salinity, and pH exerted similar effects on the relative abundance of different microorganisms (all positively or negatively), whereas temperature and TOC showed opposite effects compared to the aforementioned environmental factors (Figure 3D). Further analysis at the genus level revealed that the top ten most abundant bacterial genera belonged to the phyla *Firmicutes* (genus *Exiguobacterium*), class *Alphaproteobacteria* (genera *Sulfitobacter* and *Sphingorhabdus*), and class *Gammaproteobacteria* (including g_*Pseudomonas*, g_*Halomonas*, g_*Acinetobacter*, g_*Alcanivorax*, and g_B2M28), and others (Figure S7). The most abundant archaeal genera belonged to *Crenarchaeota* (including genera Candidatus *Nitrosopumilus*, g_*Bathyarchaeia*, and g_*Nitrosopumilaceae*), *Nanoarchaeota* (genus g_*Woesearchaeales*), and *Asgardarchaeota* (g_*Lokiarchaeia*). The top five most abundant fungal genera belonged to the phyla *Ascomycota* (including g_*Thielavia*, g_*Aspergillus*, g_*Davidiella*, g_*Verticillium*) and *Chytridiomycota* (g_*Rhizophydium*). The relative abundances of these microbial genera changed significantly beyond 75°N (Figure S7), with varying impacts of environmental factors on different genera. Latitude, salinity, and pH exerted significant effects on most genera, while only g_Ca. *Nitrosopumilus* and g_*Bathyarchaeia* were notably influenced by temperature. Additionally, the relative abundance of g_B2M28, g_*Woesearchaeales*, and g_*Rhizophydium* showed a significant positive correlation with ammonium concentrations (Figure S8).

### Distribution of the specialists across regions

**Figure 4:**
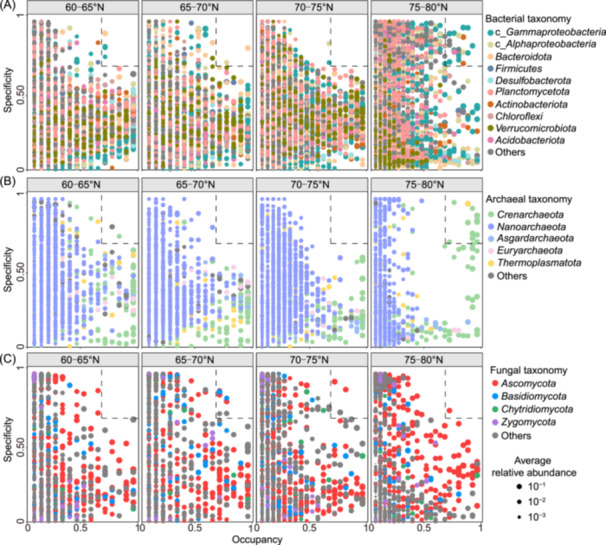
Microbial specificity and occupancy in different latitudinal regions. (A–C) Specificity–occupancy plot of bacterial (A), archaeal (B), and fungal (C) OTUs in each region are shown. The x‐axis represents occupancy, that is, how well an OTU is distributed across all sites within the same region, and the *y*‐axis represents specificity, that is, whether it is also found in other regions. OTUs with specificity and occupancy greater than or equal to 0.7 (within the dotted box) are classified as specialists (see Table S4 for the list of these specialists).

As indicated by the spread of OTUs across the x‐axis (occupancy), more bacterial and archaeal OTUs from the 75–80°N region exhibited greater variability in occupancy—fewer OTUs were consistently present across samples from this region, whereas many were widespread in the 60–75°N region. Additionally, OTUs from the 75–80°N habitat displayed significantly higher habitat specificity (Figure 4A,B). To find specialist species attributable to each latitudinal region, we selected OTUs with specificity and occupancy greater than or equal to 0.7 (dotted boxes in Figure 4), that is, they are specific to a region and common in their region in most sites. Our results demonstrated that the number of these specialist OTUs remarkably differed between regions, and remarkably higher bacterial and archaeal specialist OTUs were found in the region of 75–80°N (24 and 13 specialist OTUs for bacteria and archaea, respectively, Figure 4A,B and Table S4), followed by the region of 60−65°N (4 and 2 specialist OTUs for bacteria and archaea). However, more fungal specialist OTUs were detected in the region of 65−70°N and 70–75°N (7 and 3 specialist OTUs, respectively) rather than in other regions. Moreover, most of the bacterial specialist OTUs belonged to class *Gammaproteobacteria* (12 out of 30), followed by class *Alphaproteobacteria* (4 out of 30) and phylum *Acidobacteriota* (3 out of 30). Also, 12 *Crenarchaeota* specialist OTUs and 1 *Thermoplasmatota* specialist OTU were found in the region of 75−80°N. In addition, 3 *Ascomycota* specialist OTUs and 2 *Basidiomycota* specialist OTUs were found in the region of 65−70°N, and 2 *Ascomycota* specialist OTUs were found in the region of 70–75°N (Figure 4C).

### Microbial co‐occurrence patterns

**Figure 5:**
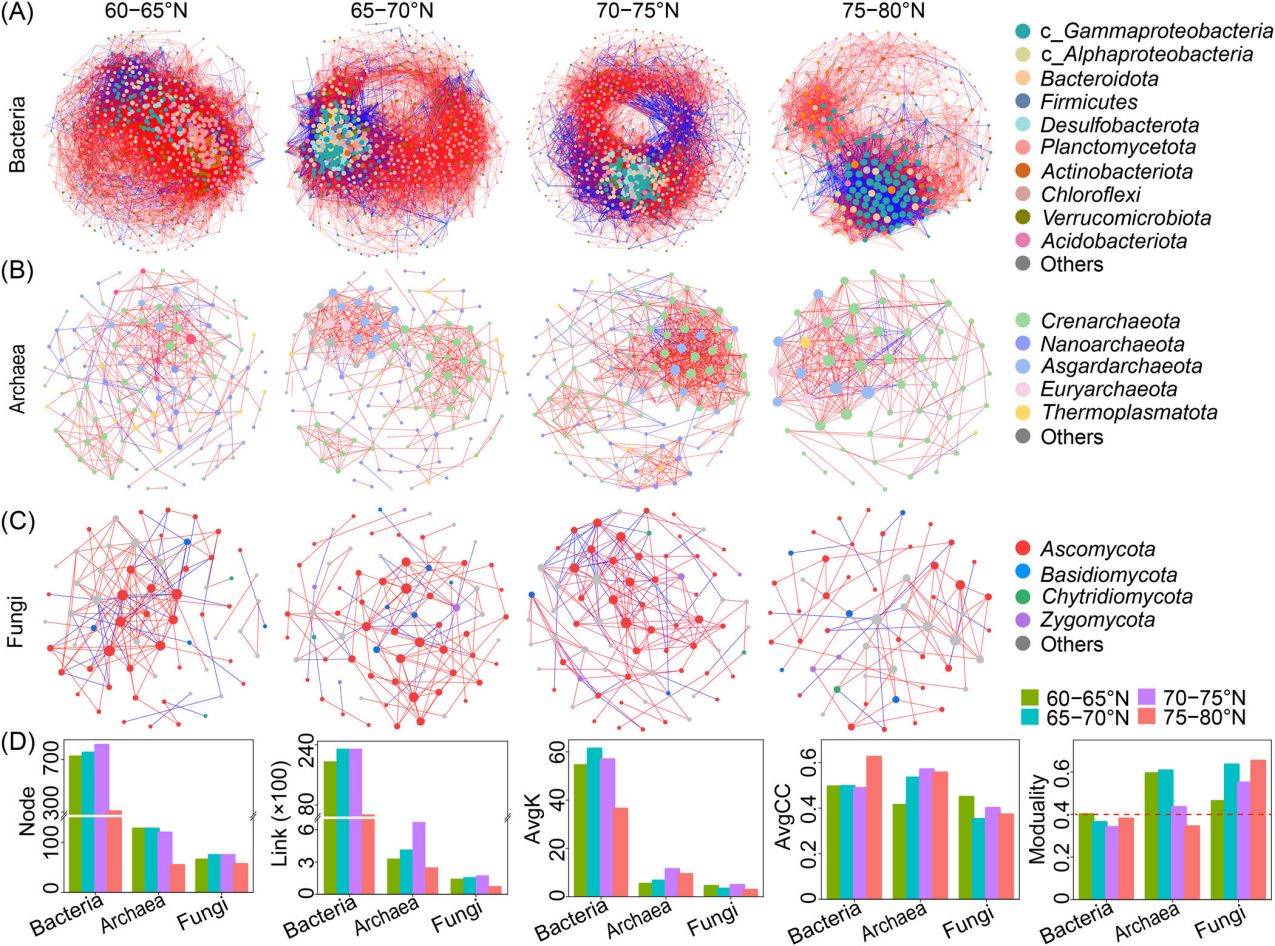
Co‐occurrence patterns of microbial communities in different geographical regions. Each node represents an individual OTU, and its size is proportional to its degree (i.e., links with other OTUs). The colors of nodes represent different microbial phyla, which are listed in the right labels. Red links indicate positive correlations and blue links indicate negative correlations. *avgK*, average degree; *avgCC*, average clustering coefficient.

Microbial co‐occurrence patterns varied significantly across different geographical regions. For bacterial communities, their co‐occurrence networks were more complex, with 304 − 809 nodes connected by 5527–22,976 links based on strong correlations (*R* > 0.7, Figure 5A). In contrast, archaeal and fungal networks had fewer nodes and links (54–127 nodes and 250–672 links for archaea, 56–74 nodes and 76–175 links for fungi, Figure 5B,C). Within these networks, bacterial networks predominantly consisted of *Gammaproteobacteria* (15.1%−31.2%), *Bacteroidota* (9.2%−11.7%), *Alphaproteobacteria* (6.5%−12.5%), *Planctomycetota* (11.2%−23.1%), and *Actinobacteriota* (5.6%–7.4%) (Figure S9A). The archaeal networks showed a stark contrast, with *Crenarchaeota* being less abundant in the 60−75°N region (41.7%−45.4%) but significantly more abundant in the 75−80°N region (81.5%). Other archaeal phyla like *Nanoarchaeota*, *Asgardarchaeota*, *Euryarchaeota*, and *Thermoplasmatota* had higher abundances in the 60–75°N region but fewer in the 75–80°N region (Figure S9B). *Ascomycota* was the dominant fungal phylum with relatively stable abundance (55.4%−60.8%), while *Basidiomycota* and *Zygomycota* were much less in the 70–75°N and 60–65°N regions, respectively (Figure S9C). Among microorganisms, positive correlations were predominant in all networks, especially in archaeal networks, where 87%–97% of the links were positive, indicating strong positive relationships within microbial communities, particularly in archaeal communities (Figure S9D). Interestingly, the complexity of microbial networks, indicated by the number of nodes and links, was the lowest in the 75–80°N region (Figure 5D). This region also showed the lowest values of average degree (*avgK*) for archaea and fungi, whereas higher clustering coefficients (*avgCC*) were observed for bacteria and archaea, but not for fungi. Modularity analysis revealed that both archaeal and fungal networks had higher modularity values, indicating a more modular structure compared to bacterial networks in Arctic sediments. This modular structure suggested a higher degree of compartmentalization within archaeal and fungal communities, potentially reflecting more specialized interactions. These findings highlighted the distinct co‐occurrence patterns of microbial communities across different regions and suggested that environmental factors and geographical locations significantly shape microbial co‐occurrence patterns.

## DISCUSSION

### Microbial diversity declines and community composition changes with latitude

Microbial ecological functions are strongly correlated with their diversity and distribution in natural systems^43^, ^56^, ^57^. Our study revealed that both microbial diversity and community similarity of bacteria and fungi exhibited lower decay rates compared to archaea in the arctic marine sediments, indicating that archaeal communities had more pronounced geographic distribution differences (Figures 1B–D and 2A–C). The lowest microbial diversity was observed in the 75–80°N region (Figures S2 and S3), consistent with earlier findings that indicated lower diversity in the Arctic Ocean (75−78°N) compared to the Bering Sea and Chukchi Sea (60−70°N)^58^, ^59^. Environmental heterogeneity is likely the key factor driving changes in microbial diversity and community composition^60^, since environmental variables like temperature, salinity, pH, and TOC significantly change with latitude and showed a significant influence on microbial diversity or community composition (Figures S1 and S4, Table S3). Our findings indicated that temperature exerted a positive impact on microbial diversity and community composition of bacteria, archaea, and fungi (Figure S4, Table S3). This is because in cold environments like Arctic sediments, even slight increases in temperature can significantly alter the biochemical processes of microorganisms, such as enzyme activity and membrane fluidity^61^. This can lead to shifts in the dynamics among microbial taxa, favoring species that can rapidly adapt to warmer conditions. In addition to temperature, salinity showed a significant negative influence on microbial richness and community composition (Figure S4, Table S3), indicating that it is an important environmental stressor in marine sediments. Our results indicated that the salinity in sediment was much higher in the 75–80°N region (Figure S1), resulting in much lower microbial diversity (Figure S3). The phenomenon of microbial niche differentiation driven by salinity changes has also been observed in the Baltic Sea^62^. Microbial responses to salinity variation are likely attributed to the differing abilities of various microbial groups to tolerate osmotic pressure changes induced by salinity fluctuations^63^, ^64^. Other factors, such as NH_4_^+^ and TOC, are significantly positively correlated with microbial diversity and community composition (Figure S4 and Table S3). This may be attributed to NH_4_^+^ and TOC serving as essential nitrogen and carbon sources, which are crucial for microbial growth and reproduction^65^, ^66^, ^67^. Additionally, VPA results indicated that environmental factors contributed more to microbial community variation than spatial factors (Figure S6). However, the impact of spatial and environmental factors on microorganisms varies across specific taxa. For instance, we found that latitude and depth had a significant negative effect on bacterial and archaeal diversity, respectively, but did not impact fungal diversity (Figure S4). In contrast, temperature showed a significant positive effect on fungal diversity, but no such effect was observed for bacteria and archaea. These contrasting patterns suggest that bacteria, archaea, and fungi may show distinct responses to spatial and environmental factors, potentially due to their differing ecological roles, adaptive strategies, or physiological requirements. Furthermore, different microbial taxa responded differently to these factors. For example, *Gammaproteobacteria* was significantly influenced by latitude, depth, salinity, and temperature, leading to its increasing relative abundance with latitude (Figure 3A,D). However, *Bacteroidota*, *Desulfobacterota*, *Verrucomicrobiota*, and *Euryarchaeota* showed trends that were completely opposite to those of *Gammaproteobacteria* (Figure 3D). These divergent patterns may be driven by ecological niches, metabolic pathways, and specific environmental tolerances of these microbial groups. Although some environmental factors are correlated in natural settings (e.g., changes in salinity and temperature with increasing latitude and depth), the variance inflation factor (VIF) values of all factors were below 10, indicating no significant autocorrelation when explaining microbial community variations. For instance, environmental factors like salinity, depth, and pH, which showed highly consistent trends, did not all exert significant effects on different microbial groups (e.g., *Bacteroidota*, *Acidobacteriota*, *Crenarchaeota*, and *Thermoplasmatota*; Figure 3D). Some microbial groups, such as *Alphaproteobacteria*, *Planctomycetota*, and *Asgardarchaeota*, even showed opposite effects in response to these factors.

It is important to acknowledge the limitations of using bottom seawater temperature and salinity as proxies for the sedimentary environmental conditions in our microbial–environmental correlation analysis. Sedimentary temperature and salinity may differ little from those in the overlying water due to the distinct microenvironment within the sediment. While we used measured bottom seawater temperature and salinity as surrogates for sediment conditions, we recognize that this approach may not fully represent the actual environmental conditions experienced by the microbes within the sediment matrix. Specifically, sediments often show slower temperature changes compared to the water column, and microbial activity may influence sediment salinity, which is not always accurately represented by measurements of near‐bottom seawater conditions. Therefore, although this proxy approach provided a more feasible and standardized environmental dataset across the sampling sites, we must consider that the true sediment conditions might differ, potentially leading to an under‐ or overestimation of environmental impacts on microbial communities. Future studies that incorporate direct measurements of sediment properties, such as porewater chemistry and sediment temperature profiles, will be essential for more accurately assessing the influence of these factors on microbial diversity and distribution.

Microbial diversity and community composition changed significantly in the 75–80°N region compared to other regions (Figures 3 and S3, Table S2). There are several potential reasons for this phenomenon. First, the 60–75°N region is much shallower (Figure S1A) compared to the 75−80°N region, facilitating the dispersal of microorganisms with marine currents in the shallow region, and shallow waters allow for more mixing and nutrient exchange, which can enhance microbial growth and diversity^68^. Second, these regions experience increased commercial operations, such as merchant shipping and fishing activities. Increased ship traffic can introduce invasive species and pollutants and disrupt local ecosystems, promoting microbial dispersal and distribution in these regions. In contrast, the Arctic Ocean (over 75°N) is farther from the mainland and experiences fewer direct human impacts. This region is less influenced by freshwater runoff and pollutants from land‐based sources, and commercial activities are relatively minimal due to harsh environmental conditions and ice cover. As a result, microbial communities in high‐latitude regions may primarily be influenced by environmental factors such as lower temperature, greater water depth, and higher salinity^69^. Our results showed that *Gammaproteobacteria* had higher relative abundance and widespread distribution in the 75–80°N region (Figures 3A and 4A), with significant positive correlations with depth and salinity, and a negative correlation with temperature (Figure 3D). *Gammaproteobacteria*, including genera like *Colwellia*, *Pseudoalteromonas*, *Psychromonas*, and *Marinobacter*, are known for their adaptability to cold, high‐salinity, and nutrient‐poor conditions due to specialized features such as flexible cell membranes, cold‐active enzymes, and efficient nutrient uptake systems^70^, ^71^, enabling them to survive in the polar marine environment. Similarly, *Crenarchaeota* play a key role in ammonia oxidation in marine ecosystems and are also preferentially abundant in deep‐sea environments^72^, ^73^. In addition, previous studies proposed that the differences in microbial diversity between shallow and deep habitat communities are attributable to (i) rapid speciation at the shallow region as a consequence of dynamic surface conditions, in contrast to (ii) spatially and temporally constant environmental conditions over longer time scales in the deep sea^74^. Therefore, the distinct microbial distribution patterns between the 60−75°N and 75−80°N regions may be related to variations in environmental conditions and differences in microbial adaptation capabilities.

### Archaeal community shows a faster distance–decay rate than bacterial and fungal communities

The distance–decay relationship (DDR), describing the decline of community similarity with increasing geographic distance, is a widely accepted ecological pattern^75^. Our results indicated that the archaeal community showed a stronger DDR pattern than the bacterial and fungal communities (Figure 2). This aligns with earlier studies indicating higher spatial turnover rates for prokaryotic than fungal communities in brackish‐saline groundwater^76^. Specifically, we found that *Crenarchaeota* showed the highest community turnover, with a distance–decay rate of −0.013 (Figure S10), followed by *Chloroflexi* (slope = −0.009) and *Desulfobacterota* (slope = −0.008). In contrast, *Firmicutes* (slope = −0.002), *Ascomycota* (slope = −0.001), *Basidiomycota* (slope = −0.002), and *Zygomycota* (slope = 0.002) showed the lowest distance–decay rates in community similarity. This variability might be due to differences in environmental adaptations among different microbial taxa^77^. For example, the faster DDR of archaea, particularly *Crenarchaeota*, suggests a higher degree of spatial turnover and environmental sensitivity compared to other microbial groups. This observation may indicate that archaea are more responsive to localized environmental factors such as temperature, salinity, and nutrient gradients, which vary significantly across the Arctic. Given the harsh and dynamic nature of Arctic ecosystems, archaea, particularly those involved in nitrogen cycling (e.g., *Crenarchaeota*), are likely highly adapted to specific niches and environmental conditions. These taxa may show a greater turnover in response to small‐scale environmental changes, reflecting their capacity to adapt to a wide range of conditions found in the Arctic, from cold temperatures to varying salinity levels. Moreover, the greater spatial turnover of archaeal communities in comparison to bacterial and fungal communities could also be related to the fact that archaea, with their unique metabolic pathways, play crucial roles in biogeochemical cycles, such as nitrogen and carbon cycling, which are highly sensitive to shifts in environmental conditions. The higher DDR in archaeal communities may, therefore, indicate a specialized adaptation to these conditions, with different archaeal populations occupying specific, geographically constrained niches within the Arctic marine sediments. We also found that few environmental factors could exert a significant influence on the fungal community, but more environmental factors showed a significant influence on the archaeal community (Table S3). Additionally, the genomic, proteomic, and evolutionary differences among the three life domains^78^ likely contribute to their difference in environmental adaptation abilities. For example, prokaryotes, including bacteria and archaea, show high genomic flexibility due to frequent horizontal gene transfer (HGT)^79^, ^80^, which enables rapid adaptation to environmental conditions. Additionally, their proteomic versatility allows them to thrive in diverse and extreme environments, with unique protein expression tailored to such conditions^81^, ^82^. In contrast, fungi show more specialized proteomes and slower evolutionary rates due to their complex cellular structures and longer generation times^83^, ^84^, ^85^, leading to more stable and less variable community compositions.

We found that the species identified as specialists among bacteria and archaea were mostly *Proteobacteria* (mainly class *Gammaproteobacteria*) and *Crenarchaeota*, and they were abundantly present in the 75–80°N region (Figure 4A,B, Table S4). This may be related to their strong adaptation, which may result in their high relative abundance^86^, ^87^, ^88^. Many members of *Crenarchaeota* possess the *amoA* gene, enabling them to oxidize ammonia to nitrite^65^, ^89^, ^90^, ^91^, ^92^. Besides, some nitrifying–denitrifying *Gammaproteobacteria* also play key roles in driving nitrogen cycling in marine ecosystems^93^. Therefore *Crenarchaeota* and *Gammaproteobacteria* are likely key drivers of nitrogen cycling in polar marine sediments. In contrast, fungal species are generally distributed homogeneously across different regions and sites, indicating their weaker habitat bias compared to bacterial and archaeal communities. Notably, the widespread and high relative abundance of genus *Myrothecium* in the 65−70°N region suggests its potential ecological functions in the local environment. *Myrothecium* is known to produce extracellular enzymes, such as cellulases and hemicellulases, which are involved in the breakdown of complex organic matter, facilitating nutrient cycling^94^, ^95^, ^96^, ^97^. This promotes nutrient cycling, ensuring the continuous turnover of organic material, maintaining soil health, and supporting the local ecosystem. Thus, the presence and activity of *Myrothecium* in Arctic marine sediments have significant ecological functions and implications for organic matter degradation and sustaining nutrient cycling in the Arctic ecosystem.

### Microbial co‐occurrence patterns change with geographical regions

Correlation‐based network analysis effectively infers potential interactions within complex microbial communities^50^, ^98^. Our findings demonstrated distinct co‐occurrence networks for bacteria, archaea, and fungi across different geographical regions. Bacterial and archaeal networks showed higher species diversity, indicated by more nodes and links, compared to fungal networks, which had fewer nodes and links (Figure 5D). This discrepancy is attributed to the generally higher abundance and diversity of bacteria and archaea (Figures 1 and S3). Moreover, higher *avgCC* values for bacteria and archaea indicated more frequent connections than those observed in fungal populations. Archaea showed more positive correlations (87%–97%) compared to bacteria (73%–88%) and fungi (72%–82%), indicating a greater tendency for cooperation to adapt to the environment. Fungal networks generally showed higher modularity than bacterial and archaeal networks, particularly in the 75–80°N region (Figure 5D), suggesting that fungal populations form more modules to maintain community stability and resist environmental perturbations^99^, ^100^. Regional variations in microbial communities were also evident (Figure S9). The higher abundance of *Gammaproteobacteria*, *Crenarchaeota*, and *Ascomycota* in co‐occurrence networks across all regions may be due to their key ecological functions. For example, *Gammaproteobacteria* participate in nitrogen and sulfur cycles, performing denitrification, nitrogen fixation, and sulfur oxidation^86^, ^87^, ^101^. *Crenarchaeota* play a key role in oxidizing NH_4_^+^ to NO_3_^−^ ^102^, and are crucial for nitrogen cycling in marine sediments. *Ascomycota* excels in decomposing complex organic matter, aiding nutrient cycling and organic matter turnover. These roles are vital for maintaining biogeochemical cycles and ecosystem stability in Arctic marine sediments.

In conclusion, we examined the microbial diversity and community composition of bacteria, archaea, and fungi across a latitudinal gradient in Arctic marine sediments. Our findings revealed a significant decline in microbial diversity and community similarity, particularly in the 75–80°N region. Environmental factors, including temperature, salinity, and TOC, played critical roles in shaping microbial communities. Additionally, the archaeal community showed the highest distance–decay rate, suggesting stronger spatial turnover compared to bacteria and fungi. Co‐occurrence network analysis further highlighted the differences in microbial interactions, with archaeal community showing more positive correlations than bacterial and fungal communities. These results underscored the importance of environmental heterogeneity in shaping microbial diversity and revealed that the response of microorganisms to the environment varied between taxa in polar regions.

## MATERIALS AND METHODS

### Sediment sampling, collection and physicochemical property analyses

A total of 72 stations were sampled from the Bering Sea to the Arctic Ocean (60–80°N, 155°–180°W); the sampling stations are shown in Figure 1A. Sediment samples were obtained from each sampling site using a box corer designed to minimize disturbance (50 × 50 × 65 cm). Following retrieval, the upper 5 cm of sediment was collected and placed in sterile tubes, rapidly flash‐frozen in liquid nitrogen, and stored at −80°C until DNA extraction.

It is important to acknowledge that in situ temperature measurements within the sediments during sampling pose significant challenges. Given the lack of hydrothermal vents and cold seeps in the sampling area, we used the temperature, depth, and salinity data from the bottom seawater, measured by conductivity–temperature–depth (CTD) oceanic profilers, as proxies for these sedimentary parameters. The pH was measured in the laboratory using a 1:2.5 ratio of freshly thawed sediment to water, using a Mettler‐Toledo FE20 pH meter (Mettler‐Toledo Instruments Co.). Additionally, approximately 10 g of sediment was dried and ground into a fine powder using a Hard Tissue Homogenizer (VWR International, West Chester, PA, USA) for other geochemical analyses according to our previous studies.^59^, ^103^ Briefly, the concentrations of NO_3_^−^ and NH_4_^+^ were measured using a FIAstar 5000 Analyzer (Foss Tecator, Hillerød, Denmark), while the TOC was analyzed using a carbon–nitrogen analyzer (Multi N/C 3000; Analytik Jena). Detailed information on the sampling locations and physicochemical parameters can be found in Table S1.

### DNA extraction, sequencing, and data processing

The methods for DNA extraction, PCR amplification, and data analysis are detailed in our previous study^59^. Briefly, DNA was extracted from 0.5 g of surface sediments using the MoBio PowerSoil DNA Isolation Kit (MoBio Laboratories, Inc.). For each sample, triplicate DNA extractions were combined. Microbial communities were characterized by amplifying a region of the 16S rRNA gene, targeting both bacteria and archaea. PCR amplification was carried out using the primer pair of 338F (5′−ACTCCTACGGGAGGCAGCA‐3′) and 806 R (5′−GGACTACHVGGGTWTCTAAT−3′) for bacteria^104^, and Arch519F (5′−CAGCMGCCGCGGTAA−3′), Arch915R (5′−GTGCTCCCCCGCCAATTCCT−3′) for archaea^105^, ITS3_KYO2 (5′−GAT GAAGAACGYAGYRAA−3′) and ITS4 (5′–TCCTCCGCTTATTGATATGC−3′) primers were used to amplify the fungal ITS2 region^106^.

Sequencing was performed on the MiSeq platform (Illumina Inc.) using paired‐end 2 × 250 bp (PE250) sequencing. The raw sequencing data were processed and analyzed using an in‐house pipeline (https://dmap.denglab.org.cn/) as described in a previous study^59^, ^107^. Sequences with an average quality score under 20 and those shorter than 200 bp were removed from the dataset. The sequences were clustered into OTUs with a 97% similarity threshold. Taxonomic assignments for bacterial and archaeal representative sequences were made using the SILVA reference database^108^, while fungal representative sequences were assigned to their respective taxonomic lineages using the Warcup database (V2 release).

### Statistical analysis

To equalize efforts, sequences were randomly resampled to match the smallest read count among all samples (39,869, 13,660, and 23,887 sequences for bacteria, archaea, and fungi, respectively). Microbial α‐diversity was estimated using richness (i.e., OTU number) and the Shannon index in this study. The overall differences in microbial community composition were visualized using NMDS analysis, performed using the “vegan” package in R software (Version 4.0.1), based on the Bray–Curtis distance. Community composition dissimilarities were assessed using three methods: permutational multivariate analysis of variance (PERMANOVA), Multi‐Response Permutation Procedures (MRPP), and Analysis of Similarities (ANOSIM).

CCA with Monte Carlo permutation tests was performed using the OTU table (via the “vegan” package in R) to investigate the impact of environmental factors on microbial community heterogeneity. Before CCA, VIFs were calculated to identify potential collinearity among environmental variables using the vif.cca function in the “vegan” package^109^. Environmental variables with VIF values greater than 10 were removed to avoid multicollinearity, and forward selection was applied to identify the explanatory variables for further analysis.

To exclude spatial autocorrelation of environmental variables and assess the independent effects of environmental factors, VPA was conducted to quantify the influence of environmental factors (E), spatial factors (S) (including longitude, latitude, and depth), and their combined effect (S&E) on community variation, using the ‘vegan’ package. Additionally, to explore the relationships between environmental variables and microorganisms, Spearman's rank correlation was applied to examine the correlations between the main microbial phyla and genera (top 10 for bacteria and top 5 for archaea and fungi) and environmental variables.

To examine how microorganisms from each region are distributed across sites and their specificity to a particular region, we calculated specificity and occupancy for each OTU following a previous study^110^. Specifically, specificity and occupancy were calculated using the formulas Specificity = Nindividuals_*S*,*H*_/Nindividuals_*S*_ and Occupancy = Nsites_*S*,*H*_/Nsites_*H*_. Here, specificity refers to the mean abundance of a species (*S*) in samples from a particular habitat (*H*), while occupancy represents the relative frequency of species *S* occurring in samples from habitat *H*. Nindividuals_*S*,*H*_ is the mean number of individual of OTU *S* across all samples from habitat *H* and Nindividuals_*S*_ is the sum of the mean number of individual *S* over all habitats; Nsites_*S*,*H*_ indicates the number of sites in habitat *H* where *S* is present and Nsites_*H*_ is the total number of samples in habitat *H* ^111^. These two metrics were then used as axes for generating SPEC–OCCU plots.

Co‐occurrence networks for bacteria, archaea, and fungi across different latitudinal regions were constructed based on Spearman's correlation. To enhance network visualization, only OTUs present in more than 50% of the samples were included. The Spearman's correlation coefficients were calculated using the “Hmisc” and “igraph” packages in R (version 4.2.0), with correlations considered significant if the absolute correlation coefficient (|*r*|) was greater than 0.7 and the *p* value was less than 0.05. The *p* values were adjusted using the false discovery rate method (Benjamini and Hochberg‐adjusted, BH) to reduce the risk of false positives, as recommended in a previous study^112^. Additionally, topological parameters, including nodes, links, *avgK*, modularity, and *avgCC*, were calculated to characterize the network topology^49^, ^113^. All network visualizations and topological analyses were performed using Gephi software (version 0.9.2).

## AUTHOR CONTRIBUTIONS

**Jianxing Sun**: Data curation (lead); formal analysis (lead); software (lead); writing—original draft (lead); writing—review and editing (equal). **Hongbo Zhou**: Supervision (equal); writing—review and editing (equal). **Haina Cheng**: Data curation (equal); formal analysis (equal); writing—review and editing (equal). **Zhu Chen**: Formal analysis (equal); software (equal); writing—review and editing (equal). **Yuguang Wang**: Funding acquisition (lead); methodology (equal); project administration (lead); resources (lead); supervision (lead); writing—review and editing (equal).

## ETHICS STATEMENT

No animals or humans were involved in this study.

## CONFLICT OF INTERESTS

The authors declare no conflict of interests.

## Supporting information

Supporting information.

Supporting information.

## Data Availability

All amplicon raw sequencing data used in this study have been submitted to the NCBI Sequence Read Archive (SRA) with BioProject accession No. PRJNA1164985.
