# Supplementary material for: Archaea show different geographical distribution patterns compared to bacteria and fungi in Arctic marine sediments
Source: mLife. 2025 Apr 24;4(2):205–18. doi: 10.1002/mlf2.70006 (PMC12042116; doi:10.1002/mlf2.70006)
Supplement: Supplementary file 2 — Supporting information. [file MLF2-4-205-s001.docx]

*Supplementary Figures of the article:*

**Archaea show different geographical distribution patterns compared to bacteria and fungi in Arctic marine sediments**

Jianxing Sun^a^, Hongbo Zhou^a,b^, Haina Cheng^a,b^, Zhu Chen^a,b^, Yuguang Wang^a,b,*^

^a^ School of Minerals Processing and Bioengineering, Central South University, Changsha, 410083, Hunan, P. R. China

^b^ Key Laboratory of Biohydrometallurgy of Ministry of Education, Changsha, 410083, Hunan, P. R. China

^*^ Corresponding author: Yuguang Wang ([ygwang@csu.edu.cn](mailto:ygwang@csu.edu.cn))

**Keywords:** Marin microorganisms; Biogeography; Spatial distribution; Co-occurrence pattern; Arctic Ocean

**This additional information contains:**

- - 11 Pages
  - 10 Figures


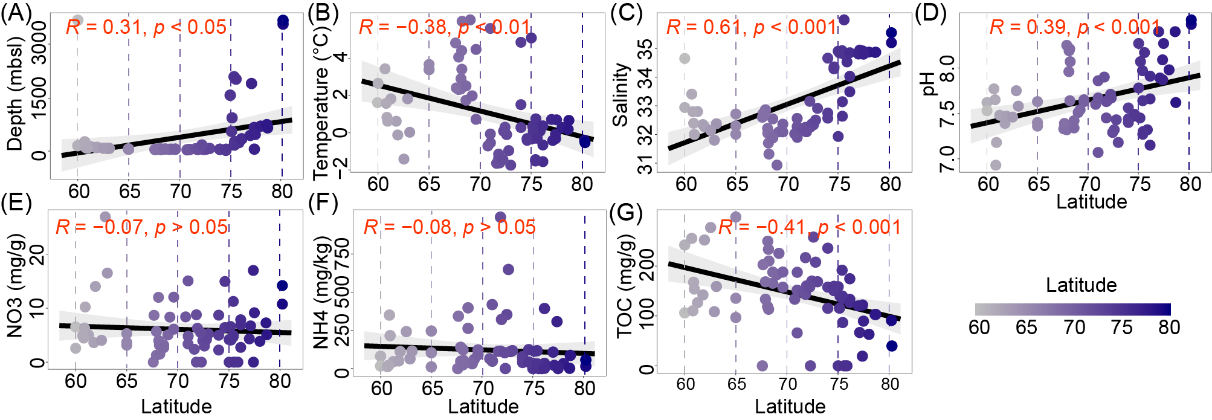


**Figure S1.** Correlation analysis of environmental variables and latitude. Linear regression lines and their 95% confidence limits (shaded gray area) are shown.


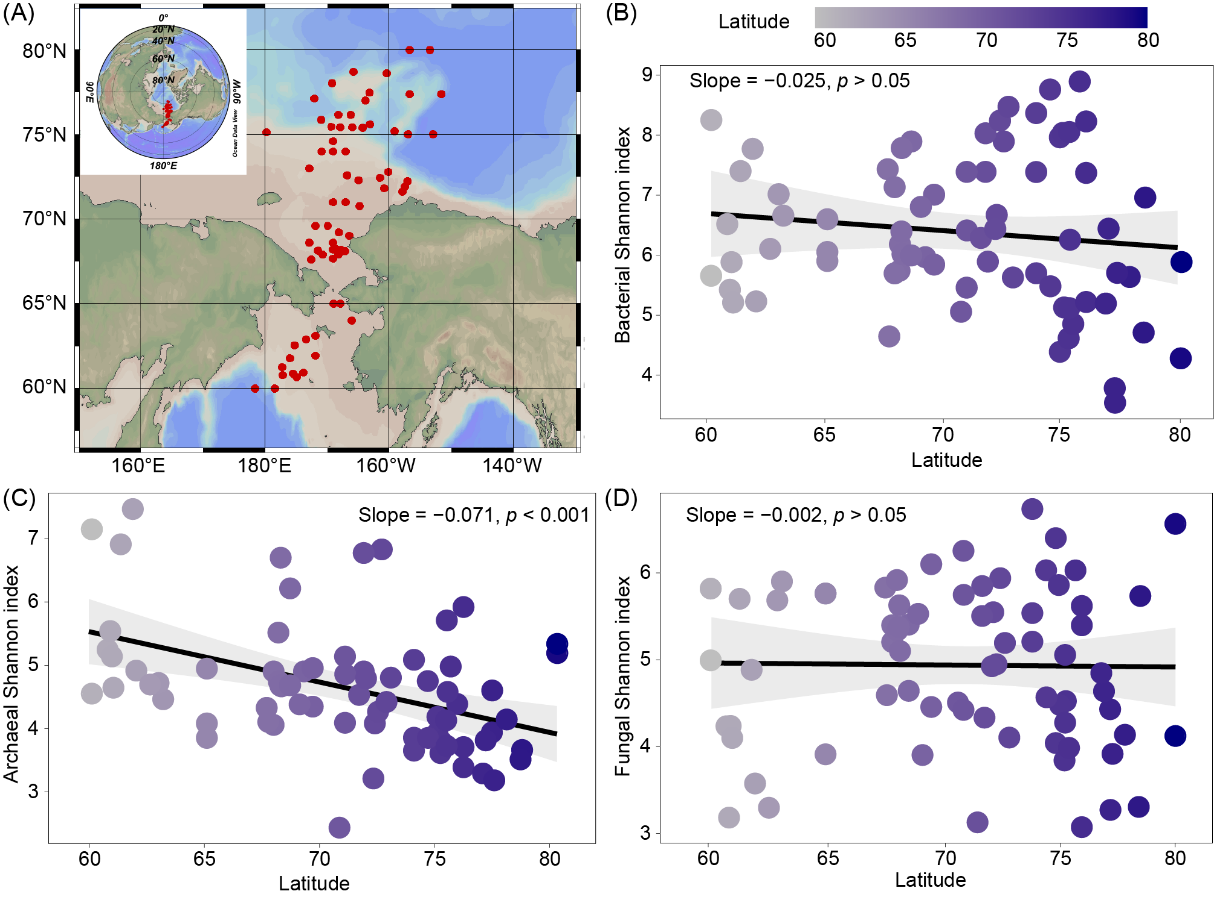


**Figure S2.** Map of sampling sites and microbial Shannon index along latitude. Linear regression lines and their 95% confidence limits (shaded gray area) are shown.


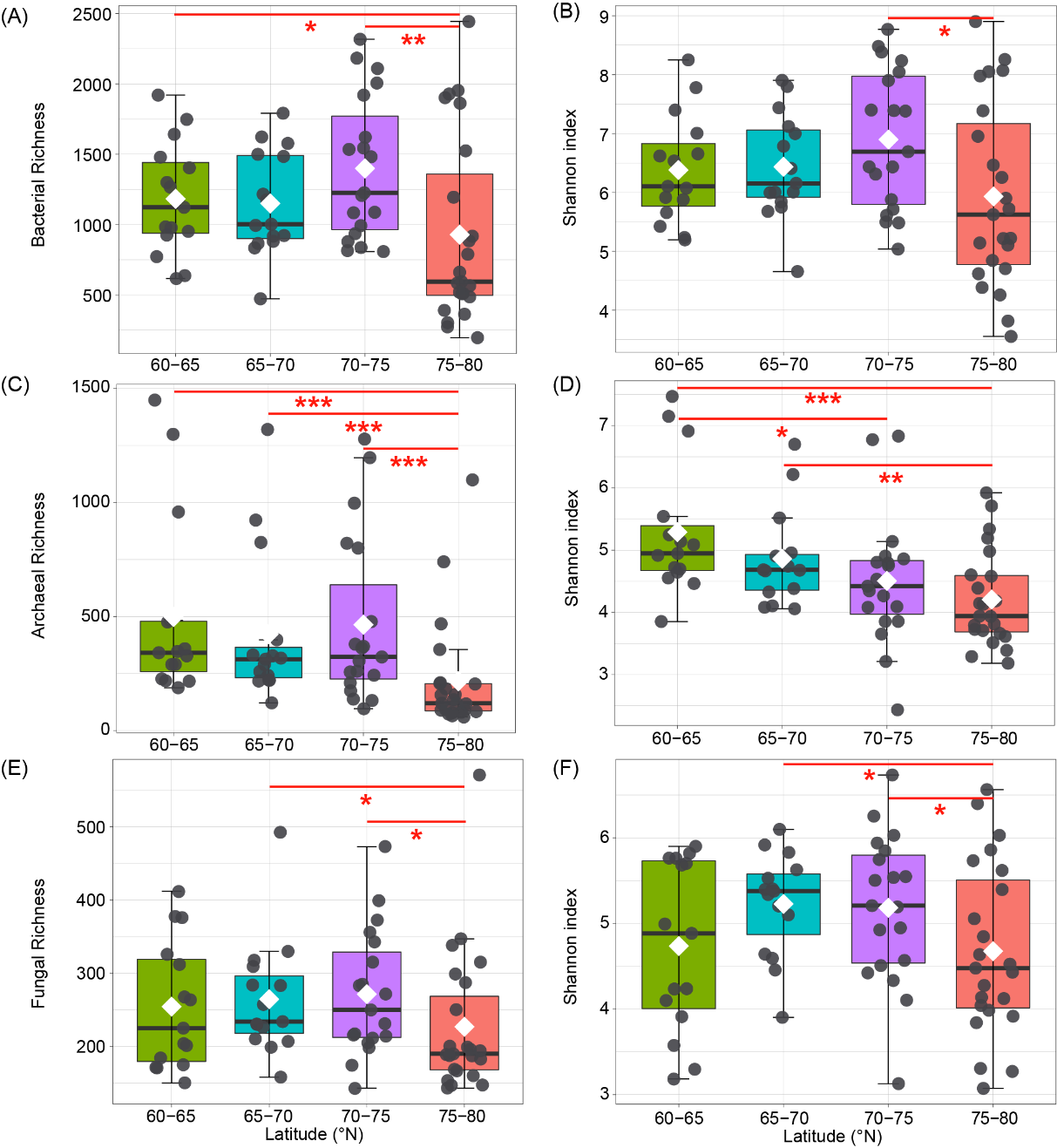


**Figure S3.** Compare of different latitudinal groups microbial richness and Shannon index based on the Wilcoxon test (*: p<0.05, **: p<0.01, ***: p<0.001).


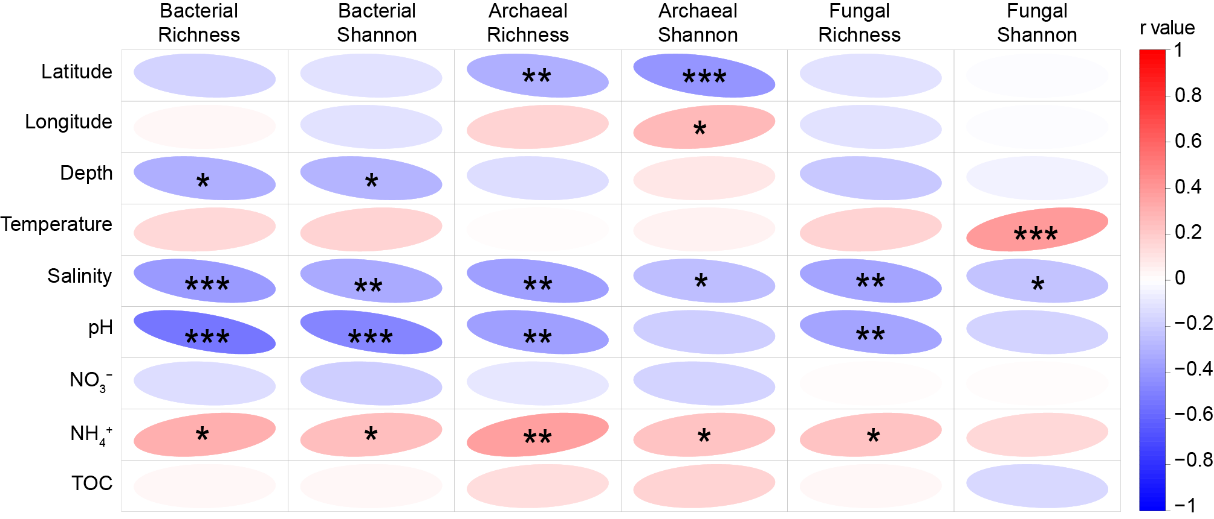


**Figure S4.** Correlation analysis of microbial diversities and environmental variables.


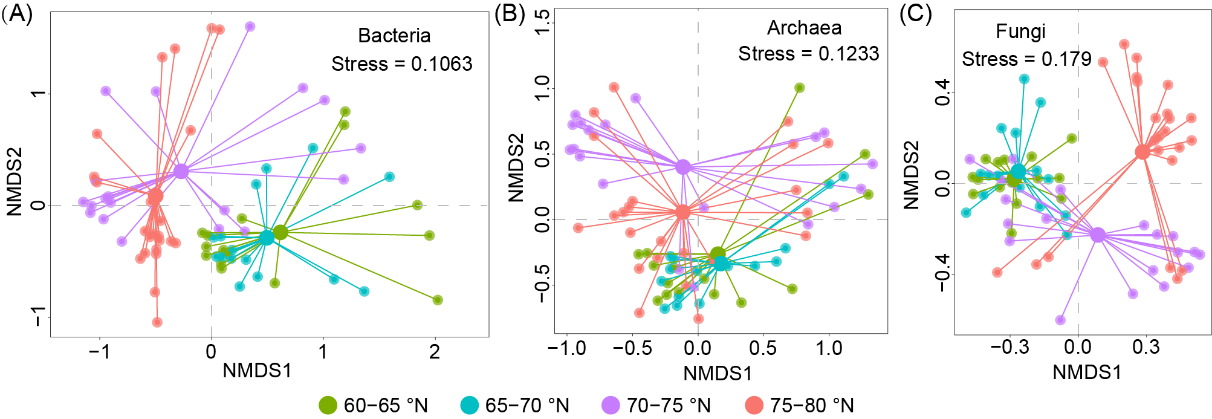


**Figure S5.** Non-metric multidimensional scaling (NMDS) analysis of bacteria, archaea, and fungi community structures based on Bray-Curtis distance.


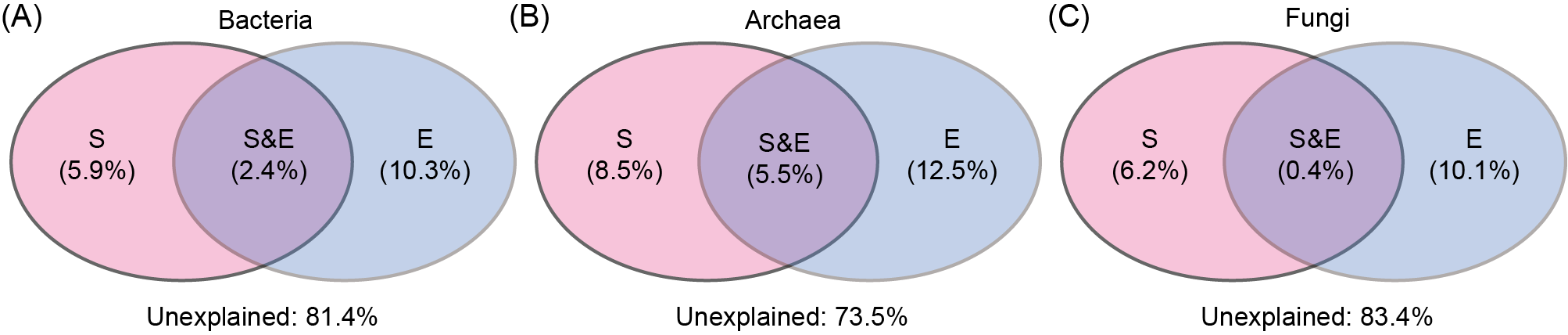


**Figure S6.** Explained proportions of variance in bacterial (A), archaeal (B), and fungal (C) communities by spatial and environmental variables based on variation partition analysis (VPA) (S: pure effect of spatial factors, including latitude, longitude, and depth; E: pure effect of environmental factors, including temperature, salinity, pH, TOC, NO_3_^-^ and NH_4_^+^; S&E: shared effect of spatial and environmental factors). The unexplained refers to the proportions of variations in community composition that cannot be explained by the two kinds of factors.


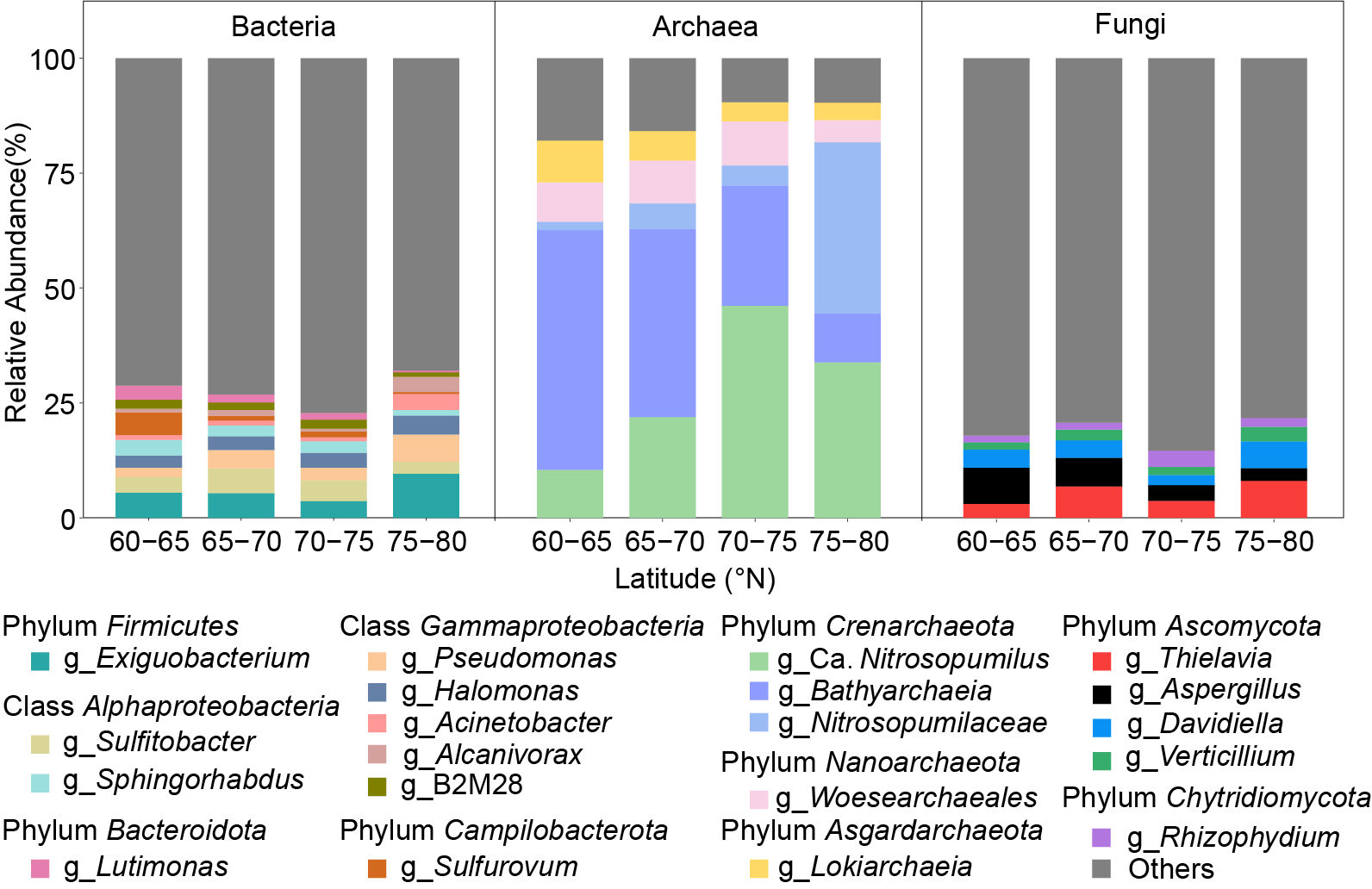


**Figure S7.** Changes in microbial abundance across different regions at the genus level.


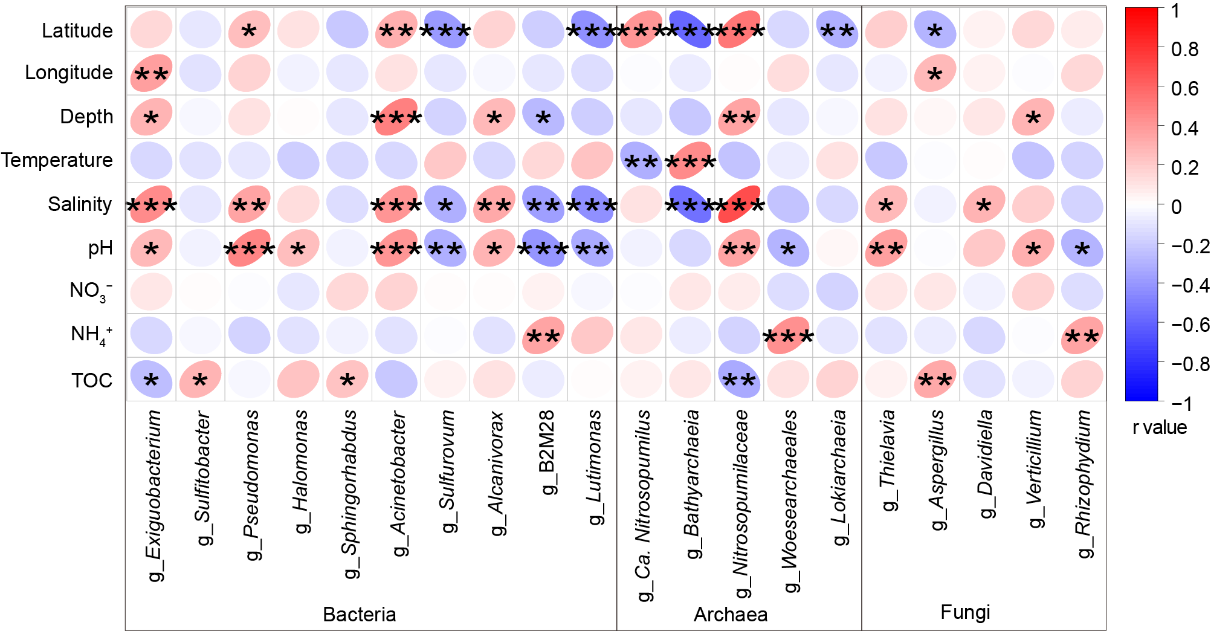


**Figure S8.** The effects of different environmental factors on the genus-level abundance of microorganisms.


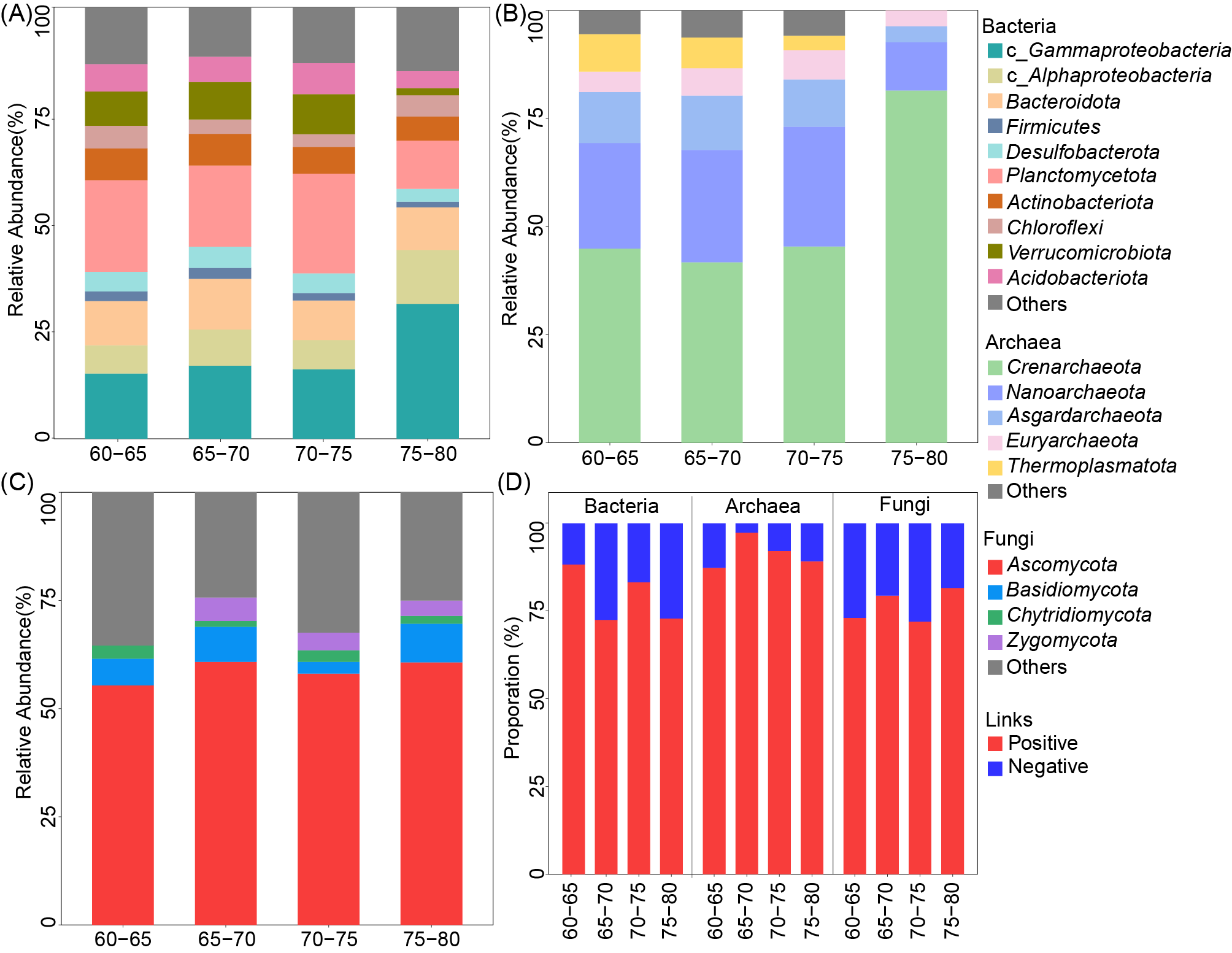


**Figure S9.** The relative abundance of major A) bacterial, B) archaeal, C) fungal microorganisms, and D) positive/negative links within co-occurrence networks.


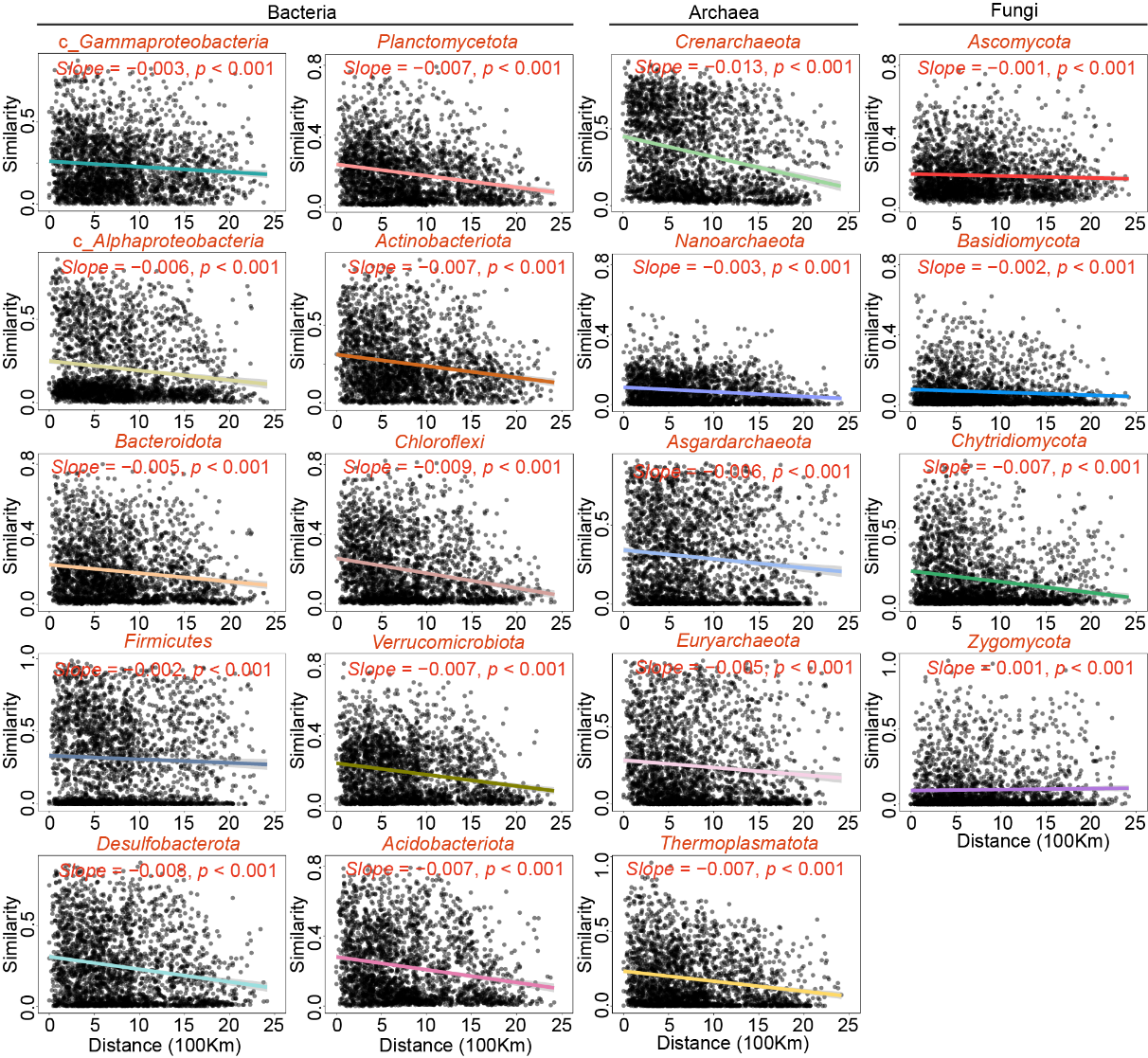


**Figure S10.** Distance decay relationship of different microbial taxa.
